# Spatiotemporal variation of climate of different flanks and elevations of the Qinling–Daba mountains in China during 1969–2018

**DOI:** 10.1038/s41598-022-10819-3

**Published:** 2022-04-28

**Authors:** Xincan Lan, Wuyang Li, Jiale Tang, Abdul Shakoor, Fang Zhao, Jiabin Fan

**Affiliations:** 1 College of Geography and Environmental Science, Henan University, Kaifeng, 475004 Henan China; 2 Key Laboratory of Geospatial Technology for the Middle and Lower Yellow River Regions (Henan University), Ministry of Education, Kaifeng, 475004 Henan China

**Keywords:** Climate sciences, Environmental sciences

## Abstract

Climate change exhibits great variation on different flanks and at different elevations in the same mountain range. To investigate the complexity of the geographic patterns of climate and phenology in the Qinling–Daba mountains (QDM), in the North–South transition zone of China, this study analyzed the spatiotemporal distribution characteristics of daily air temperature and precipitation data measured at 118 national weather stations (1969–2018). The principal findings were as follows. (1) Overall, a significant trend of warming was detected in all seasons over the past 50 years, with rates of increase of 0.347, 0.125, 0.200 and 0.302 °C/10a, in spring, summer, autumn and winter, respectively. Precipitation did not show significant variation at most stations in different seasons. (2) The rising rate of air temperature varied considerably between different flanks. Generally, air temperature change on northern flanks was greater than on southern flanks in all seasons. The tendency of air temperature change was greater in spring and winter than in summer and autumn on different flanks in the QDM. (3) The rate of increase in high-elevation regions was greater than in low-elevation regions in summer, autumn and winter, e.g., 0.440, 0.390 and 0.456 °C/10a at 3000–4000 m and 0.205, 0.218 and 0.303 °C/10a at 0–1000 m, respectively. However, in spring, the rate of increase in low-elevation regions were higher than in high-elevation regions, e.g., 0.369 °C/10a at 0–1000 m and 0.317 °C/10a at 3000–4000 m.

## Introduction

The Qinling–Daba mountains (QDM), located in the North–South transition zone and between the Tibetan Plateau and the eastern plains in China, is considered both as a natural boundary between the warm temperate zone and the north subtropical zone, and as an ecological corridor connecting the Tibetan Plateau and the eastern plains^1,2^. The climate of the QDM is accordingly characterized by complexity and diversity on different flanks and terrain^3–5^. The southern flank of the Qinling Mountains, presenting windward slopes to moisture-carrying air from the southeast, has a sunny aspect, relatively flat terrain affected by fold belts, and wet and warm climatic conditions, whereas the northern flank, presenting leeward slopes, has a shady aspect, steep terrain formed by fault belts, and relatively dry and cold climatic conditions^1,6^. Accordingly, climate change and its influence vary markedly on different flanks and at different elevations. It has been recognized that the humidification rate and climate warming are greater on the northern flank of the Qinling Mountains than on the southern flank^7,8^, but that the improvement in net primary production of vegetation is higher on the southern flank than on the northern flank at elevations below 2000 m^4,7^. However, most previous related studies focused mainly on climate change in partial regions, e.g., the Qinling Mountains^3,7,9^, Huaihe Basin^10^, and Daba Mountains^11^. Although the QDM represent an integrated geographic region connecting the warm temperate zone to the north subtropical zone, little attention has been paid to climate change within the entire region^1^. The objective of this study was to reveal the spatiotemporal variation of climate change and its complexity on different flanks and at different elevations in the QDM, to contribute to the exploration of the cause of the North–South regional differentiation in China. First, we collected and compiled air temperature data and precipitation data measured at 118 national weather stations (1969–2018). Then, we used the Manne–Kendall method to analyze the heterogeneities and similarities with climate change on different flanks and at different elevations in the QDM during 1969–2018.

## Study area

**Figure 1:**
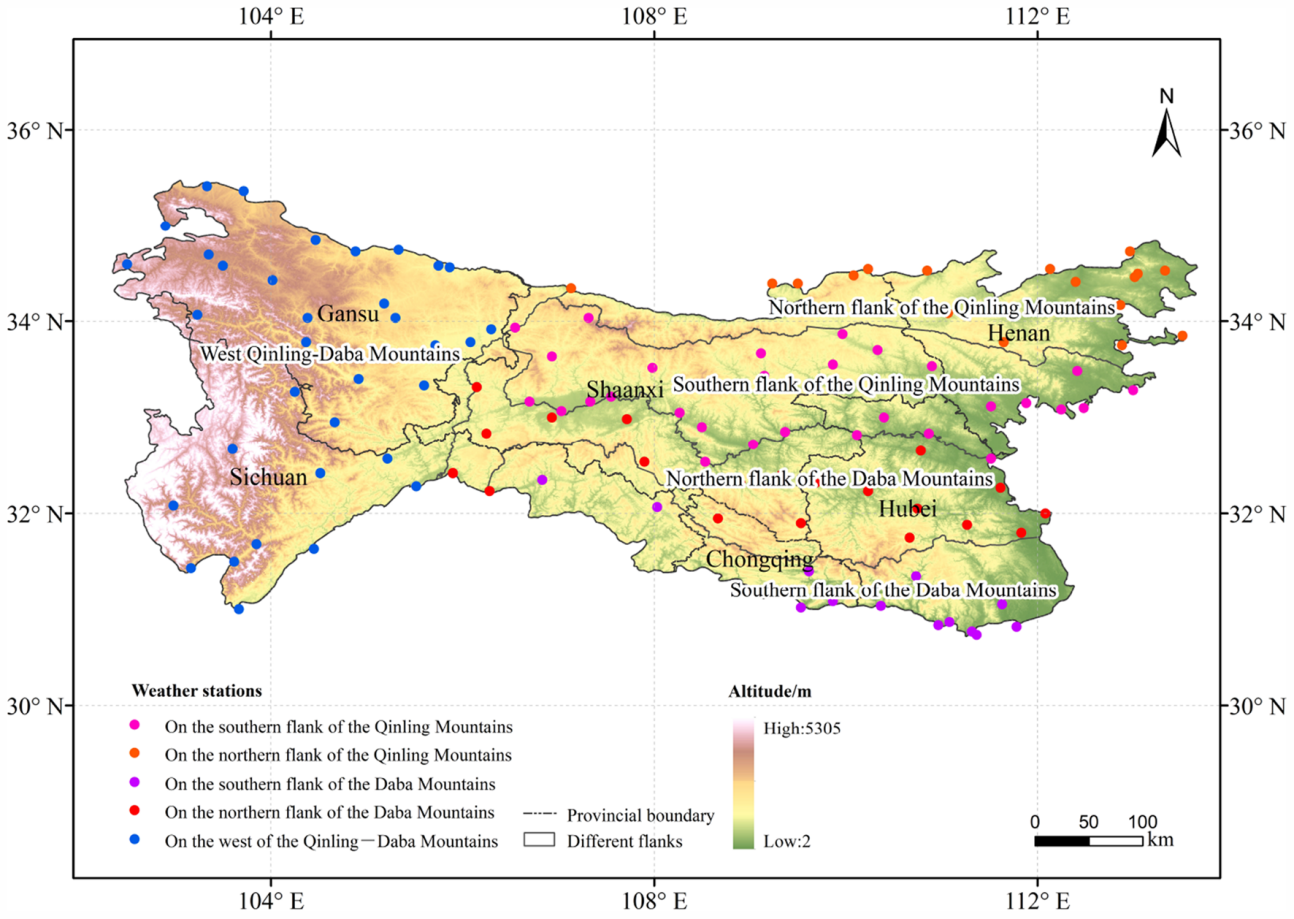
The study area and the spatial distribution of weather stations in the QDM. Pink dots represent weather stations on the southern flank of the Qinling Mountains (31), orange dots represent weather stations on the northern flank of the Qinling Mountains (20), purple dots represent weather stations on the southern flank of the Daba Mountains (13), red dots represent weather stations on the northern flank of the Daba Mountains (20), and blue dots represent weather stations on the western flank of the Qinling–Daba mountains (34). Notes: the map is created via the software ArcGIS 10.2 and the related research marks and words are added. The data of the map is from the site: https://earthexplorer.usgs.gov/, https://data.cma.cn/.

The QDM, located in central China (30°–36°N, 101°–115°E, span six provinces: Gansu, Sichuan, Shaanxi, Chongqing, Henan, and Hubei (Fig. 1). The QDM, consisting primarily of the Qinling Mountains and the Daba Mountains separated by the Han River valley, form a natural boundary between the warm temperate zone and the subtropical zone. Generally, the Qinling Mountains are broadly coincident with the 0 °C isotherm in January, the 800-mm isohyet, and the 2000-h sunshine duration contour in China^12^. Variation in these climatic factors causes the regional vegetation to change gradually from a deciduous broadleaved forest zone to an evergreen broadleaved forest zone across the QDM from north to south^13,14^. However, western parts of the QDM are dominated by cold temperate grassland because of the higher elevation and the drier climate in certain valleys^15^.

A network of 118 national weather stations is distributed over different flanks in the QDM: 31 and 20 stations are located on the southern and northern flanks of the Qinling Mountains, respectively, 13 and 20 stations are distributed on the southern and northern flanks of the Daba Mountains, respectively, and 34 stations are distributed on the western flank of the QDM (Fig. 1).

## Materials and methods

### Weather stations

A dataset comprising daily temperature and precipitation data (1969–2018) measured at 118 national weather stations in the QDM was obtained from the National Meteorological Information Center (https://data.cma.cn/), and the temperature and precipitation data were averaged by year, season and month. As shown in Fig. 1, the weather stations are mainly distributed over six provinces: Gansu, Shaanxi, Henan, Sichuan, Hubei and Chongqing.

### DEM data

For this study, digital elevation model (DEM) data for different blocks of the QDM were downloaded from the website of the U.S. Geological Survey (https://earthexplorer.usgs.gov/) and then spliced into a complete regional DEM covering the entire area of the QDM with 1000-m spatial resolution. To analyze the association between climate change and elevation, the DEM data were classified into four levels: 0–1000, 1000–2000, 2000–3000, and 3000–4000 m. The variation characteristics of the weather stations (1969–2018) were determined for the different classification segments, and the relationship between climate change and elevation specific to the QDM was explored.

### Exposure data

To decipher the association between climate change and different flanks in the QDM, we divided the QDM into five parts: the southern and northern flanks of the Qinling Mountains, the southern and northern flanks of the Daba Mountains, and the western flank of the QDM. We classified and divided the QDM on the basis of the positions of the main ridge lines and major rivers (i.e., the Jialing River and the Han River) shown in Fig. 1. The rates of change of temperature and precipitation in the five regions were calculated through statistical analysis to explore the variations and similarities of climate change on the different flanks of the QDM in the North–South transition zone of China.

### Method

The widely used Manne–Kendall method is effective in detecting change in long-term time series^16^. The details of the method of treatment are described in Xu et al.^17^ In this study, the Manne–Kendall method was applied to detect changes in the long-term trends of temperature and precipitation on different flanks and at different elevations of the QDM using the meteorological data from the 118 national weather stations.

## Results

### Air temperature changes on different flanks in the QDM during 1969–2018

**Figure 2:**
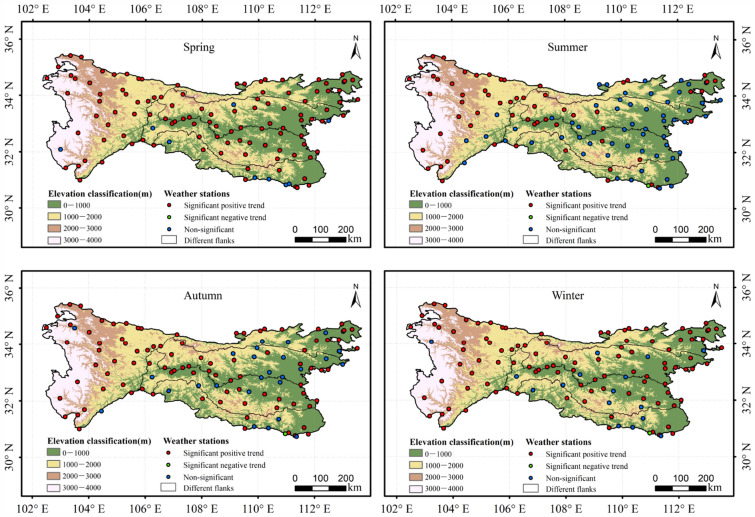
Significance analysis of mean air temperature based on the Mann–Kendall test at the 5% significance level at different weather stations in the QDM during 1969–2018. Notes: the map is created via the software ArcGIS 10.2 and the related research marks and words are added. The data of the map is from the site: https://earthexplorer.usgs.gov/, https://data.cma.cn/.

The Mann–Kendall test revealed that 108 (91.53%) of the stations presented a significant trend of increase in spring air temperature (Fig. 2), while 61 (51.69%), 91 (77.12%), and 102 (86.44%) of the stations showed a substantial trend of increase in air temperature in summer, autumn, and winter, respectively. A significant trend of increase in air temperature was shown by a greater proportion of stations on the western flank of the QDM than by stations on other flanks in different seasons. For example, 97.06%, 88.24%, 94.12%, and 97.06% of stations in the western QDM were found to have a notable trend of increase in air temperature in spring, summer, autumn, and winter, respectively, whereas such trends were detected on the other flanks at less than 89.29%, 35.71%, 69.05%, 80.95% of stations, respectively. In comparison with the southern flank of the Qinling Mountains, the northern flank had a greater proportion of weather stations showing a significant trend of increase in air temperature in different seasons. For example, 95%, 40%, 75%, and 95% of stations on the northern flank showed a significant trend of increase in spring, summer, autumn, and winter, respectively, while in the corresponding seasons, 93.55%, 35.48%, 64.52% and 87.10% of stations on the other flanks showed a significant trend of increase, respectively. On the southern flank of the Daba Mountains, 7.69% of stations showed a significant negative trend in air temperature in summer, autumn, and winter.

**Figure 3:**
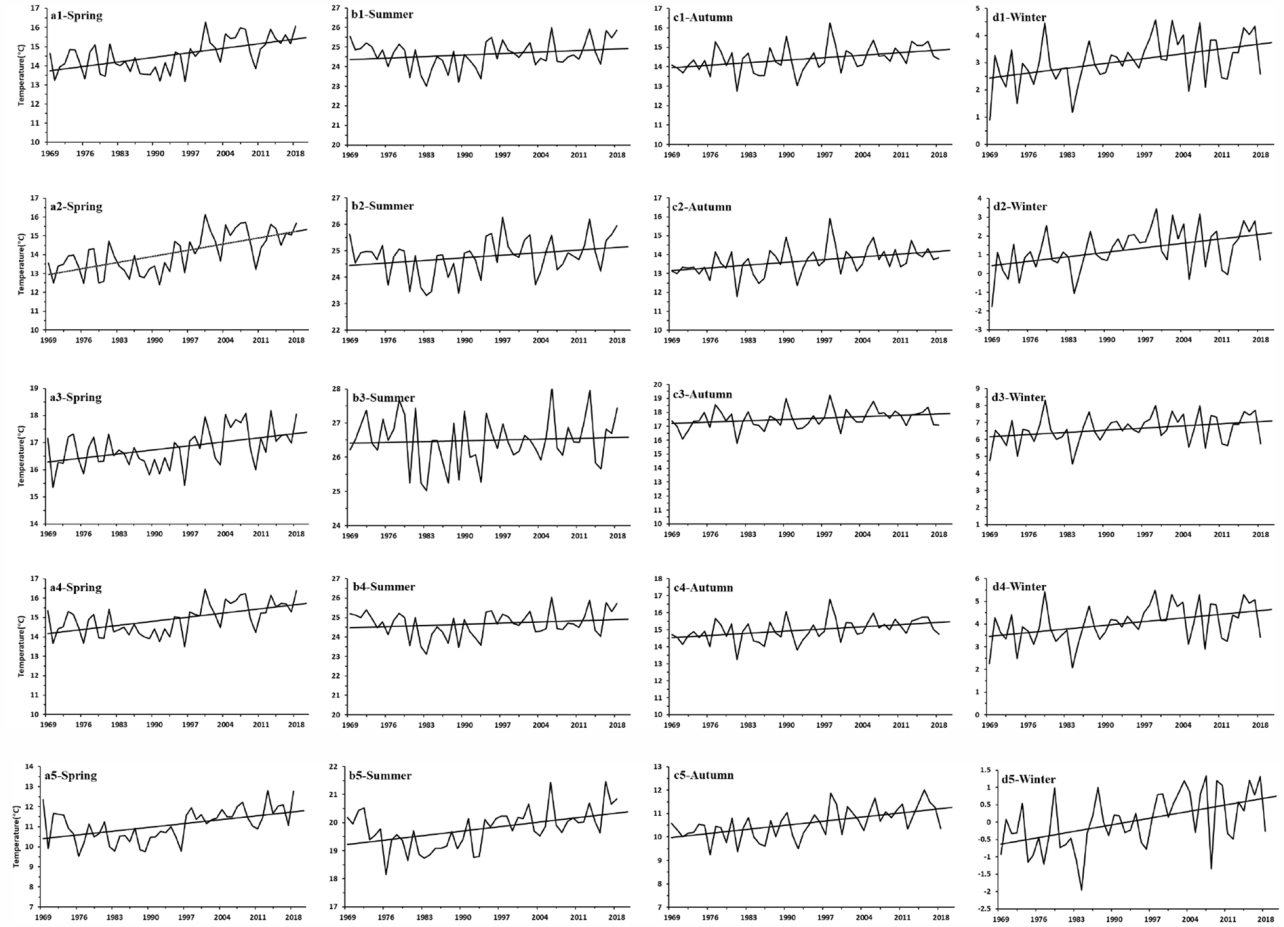
(From top row to bottom row) Variation in mean air temperature on the southern flank of the Qinling Mountains, the northern flank of the Qinling Mountains, the southern flank of the Daba Mountains, the northern flank of the Daba Mountains, and the western flank of the Qinling–Daba mountains (**a1**–**a5** spring), (**b1**–**b5** summer), (**c1**–**c5** autumn), and (**d1**–**d5** winter).

Trend analysis for the mean air temperature in different seasons revealed a significant trend of increase on the different flanks of the QDM during 1969–2018 (Fig. 3, Table 1). However, the rate of increase in air temperature in different seasons varied markedly between different flanks. The rate of increase in air temperature on the northern flank of the QDM was greater than that on the southern flank in all four seasons (Fig. 3). Specifically, the air temperature tendency on the different flanks of the QDM was greater in spring and winter than in summer and autumn. Climate warming was least in summer, i.e., the rate of increase in air temperature was as low as 0.123, 0.074, 0.085, 0.056, and 0.285 °C/10a on the northern flank of the Qinling Mountains, the southern flank of the Qinling Mountains, the northern flank of the Daba Mountains, the southern flank of the Daba Mountains, and the western flank of the QDM, respectively.

**Table 1:** Seasonal air temperature trends (°C/10a) on different flanks in the Qinling–Daba mountains during 1969–2018.

Different flanks	Spring temperature (°C/10a)	Summer temperature (°C/10a)	Autumn temperature (°C/10a)	Winter temperature (°C/10a)
Southern flank of Qinling Mountains	0.342**	0.074**	0.181**	0.267**
Northern flank of Qinling Mountains	0.479**	0.123**	0.205**	0.374**
Southern flank of Daba Mountains	0.224**	0.056**	0.112**	0.234**
Northern flank of Daba Mountains	0.338**	0.085**	0.194**	0.274**
West Qinling-Daba Mountains	0.350**	0.285**	0.308**	0.362**

**Indicates that the climate trends are significant at the 0.01 levels.

### Air temperature changes at different elevations in the QDM during 1969–2018

Statistical analysis of the trends in mean air temperature showed that stations with significant trends of increase varied markedly in different seasons and at different elevations in the QDM (Fig. 2). In the QDM, 79 (89.77%), 31 (35.23%), 61 (69.32%), and 72 (81.82%) of the stations were found to have a significant trend of increase in air temperature at elevations of 0–1000 m in spring, summer, autumn, and winter, respectively, whereas 98.68%, 92.50%, and 100% of stations were found to have a significant trend of increase at elevations of 1000–2000, 2000–3000, and 3000–4000 m, respectively. It was discovered that the area of warming in high-elevation areas was greater than that in low-elevation areas in different seasons.

**Figure 4:**
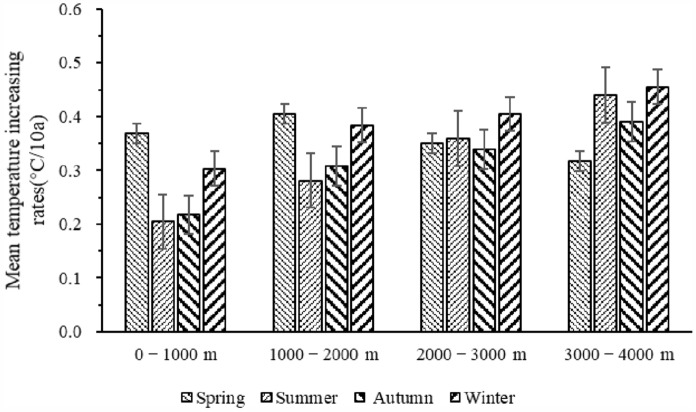
Seasonal rate of increase in air temperature at different elevations in the Qinling–Daba mountains during 1969–2018.

The air temperature trend analysis for different seasons revealed significant trends of increase at different elevations in the QDM during 1969–2018. (Fig. 4, Table 2). However, the rate of increase in mean air temperature varied greatly depending on season and elevation. At higher elevations, we detected higher rates of increase in mean air temperature in summer, autumn, and winter (Fig. 4, Table 2). For example, at elevations of 3000–4000 m, the rate of increase in air temperature in summer, autumn, and winter could reach 0.440, 0.390, and 0.456 °C/10a, respectively, while at elevations of 0–1000 m, the rate of increase was 0.205, 0.218, and 0.303 °C/10a, respectively. Conversely, the rate of increase in air temperature in spring was higher in low-elevation areas (0–1000 and 1000–2000 m) than in high-elevation areas (2000–3000 and 3000–4000 m).

**Table 2:** Seasonal rate of increase in mean air temperature (°C/10a) at different elevations in the Qinling–Daba mountains during 1969–2018.

Elevation classification (m)	Spring temperature (°C/10a)	Summer temperature (°C/10a)	Autumn temperature (°C/10a)	Winter temperature (°C/10a)
0–1000	0.369**	0.205**	0.218**	0.303**
1000–2000	0.405**	0.281**	0.308*	0.384**
2000–3000	0.351**	0.359**	0.339**	0.405**
3000–4000	0.317**	0.440 **	0.390**	0.456**

* and ** indicate that the climate trends are significant at the 0.05 and 0.01 levels, respectively, based on the Mann–Kendall test for long-term trends.

### Precipitation changes on different flanks and at different elevations in the QDM during 1969–2018

**Figure 5:**
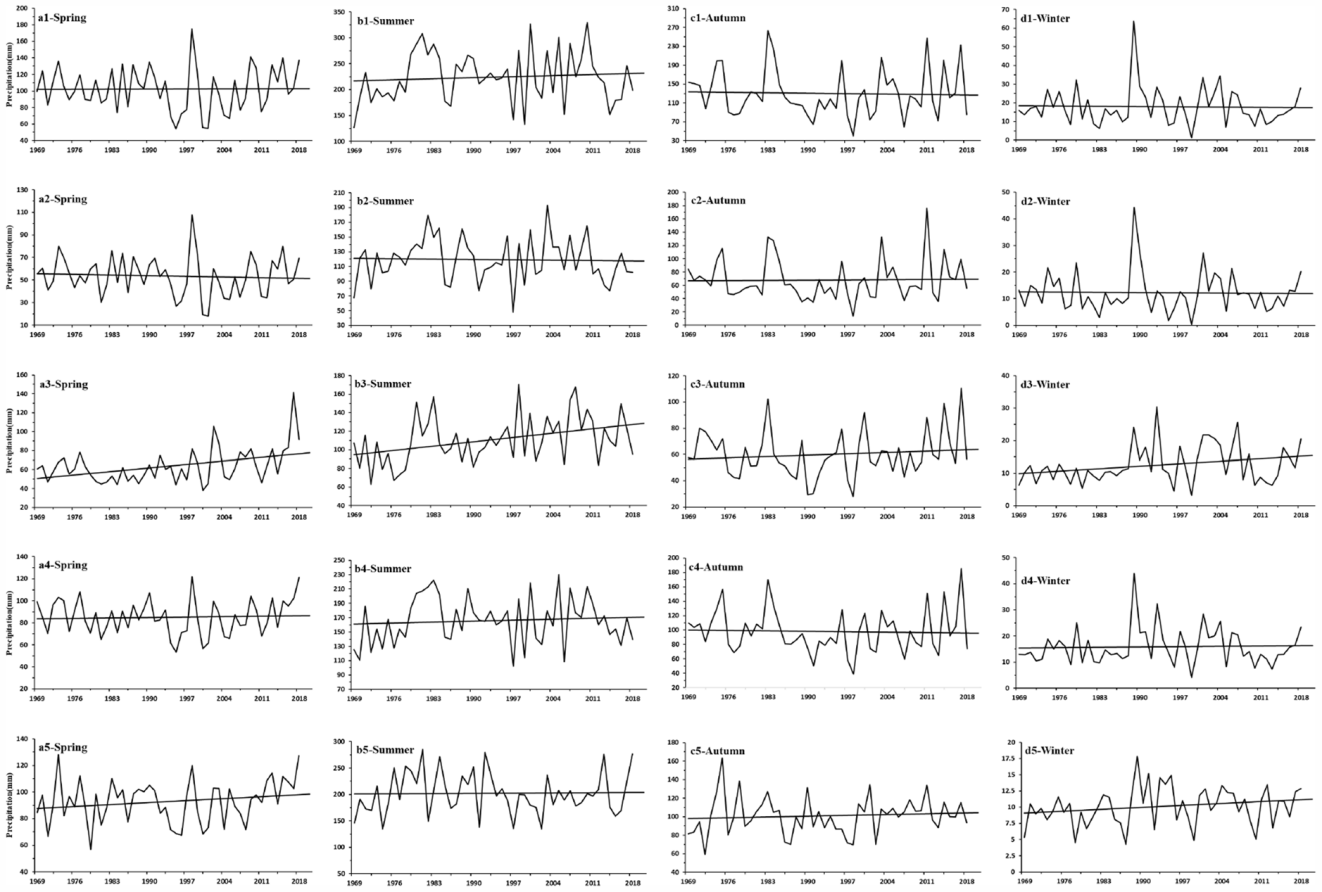
(From top to bottom) Variation in mean precipitation on the southern flank of the Qinling Mountains, the northern flank of the Qinling Mountains, the southern flank of the Daba Mountains, the northern flank of the Daba Mountains, and the western flank of the Qinling–Daba mountains (**a1**–**a5** spring, **b1**–**b5** summer, **c1**–**c5** autumn, and **d1**–**d5** winter).

**Figure 6:**
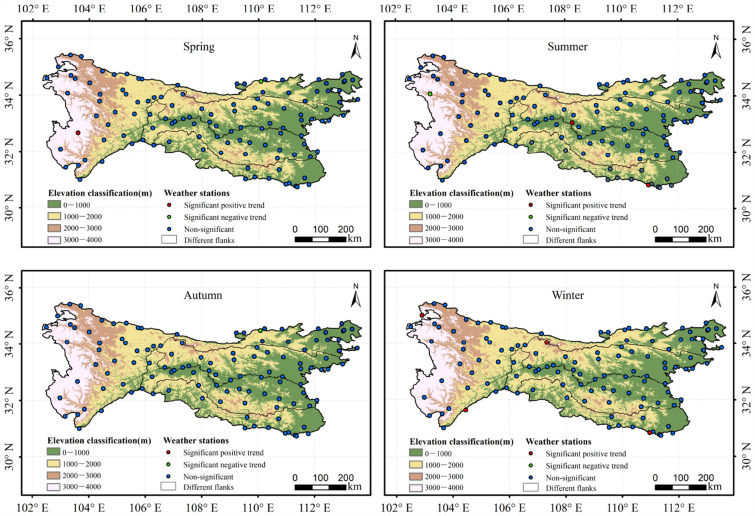
Significance analysis of precipitation changes for different weather stations based on the Mann–Kendall tests at the 5% significance level at different elevations, on different flanks, and in different seasons in the Qinling–Daba mountains during 1969–2018.

The Mann–Kendall tests did not indicate significant trends in precipitation (Fig. 5). However, some stations did exhibit notable trends in precipitation in spring, summer, and winter (Fig. 6). The weather stations are distributed at various elevations on the different flanks of the QDM. For example, Mount Hua station is in the 2000–3000 m zone on the northern flank of the Qinling Mountains, Shiquan station is in the 0–1000-m zone on the southern flank of the Qinling Mountains. Taibai station is in the 1000–2000-m zone on the southern flank of the Qinling Mountains, Zigui station is in the 0–1000-m zone on the southern flank of the Daba Mountains, and Beichuan station is in the 0–1000-m zone and Diebu, Songpan, and Hezuo stations are in the 2000–3000-m zone on the western flank of the QDM.

**Figure 7:**
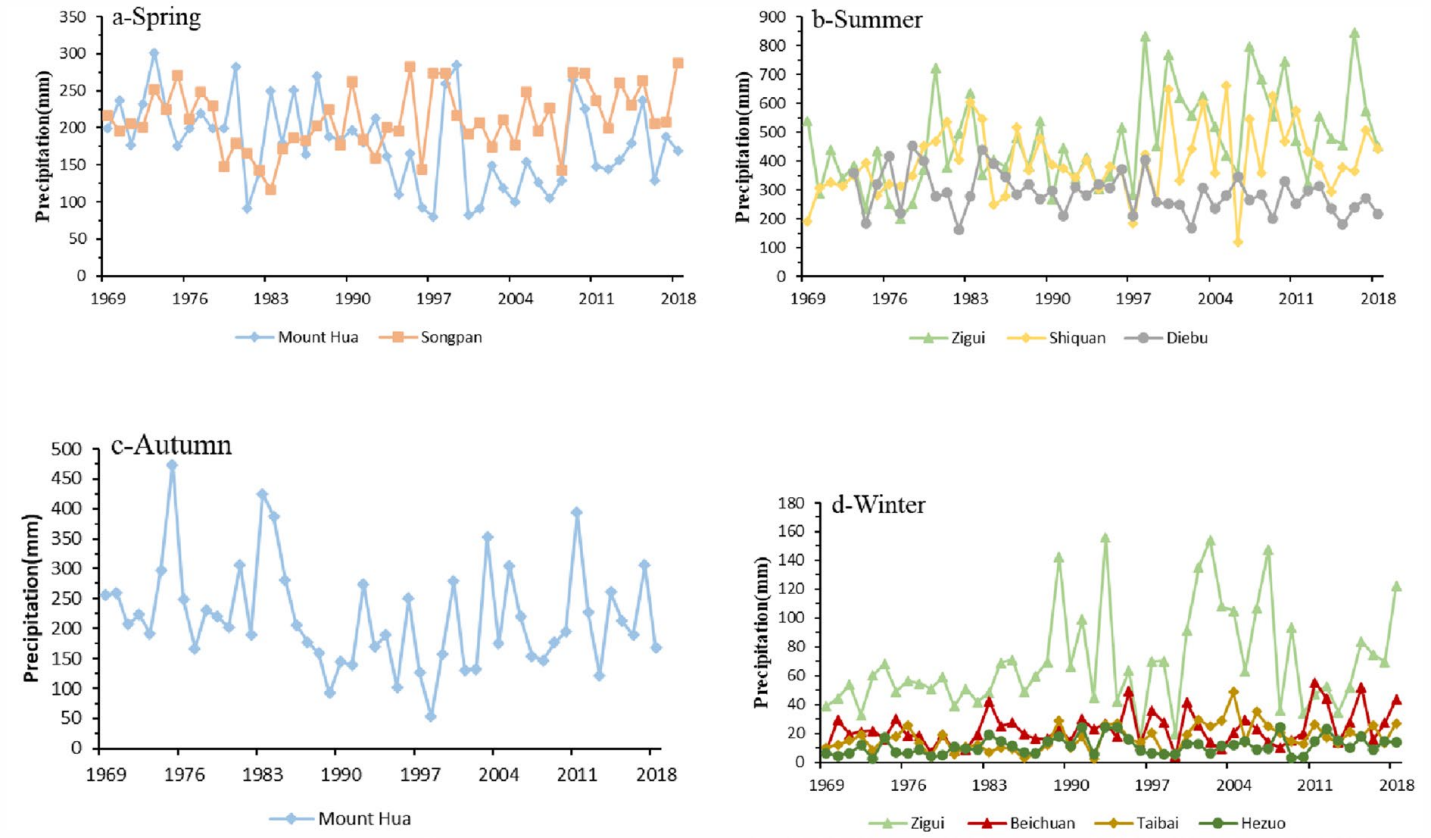
Stations with significant change in precipitation in different seasons in the Qinling–Daba mountains during 1969–2018: (**a**) spring, (**b**) summer, (**c**) autumn, and (**d**) winter.

As shown in Fig. 7 and listed in Table 3, Zigui and Shiquan stations showed significant trends of increase in summer precipitation, with change rates as high as 47.36, and 22.91 mm/10a, respectively, while one station (Diebu) showed a significant trend of decrease (− 19.43 mm/10a). In contrast, four weather stations (Zigui, Beichuan, Taibai, and Hezuo) indicated increasing changes in winter precipitation. It is important to note that Mount Hua station exhibited a significant trend of decrease in precipitation in both spring and autumn with a rate of − 14.60 and − 11.50 mm/10a, respectively. In spring, only Songpan station showed a trend of increase in precipitation, i.e., 7.63 mm/10a.

**Table 3:** Seasonal rate of change in mean precipitation (mm/10a) at weather stations with a significant trend in different seasons in the Qinling–Daba mountains during 1969–2018.

Stations name	Latitude (N°)	Longitude (E°)	Altitude (m)	Spring precipitation (mm/10a)	Summer precipitation (mm/10a)	Autumn precipitation (mm/10a)	Winter precipitation (mm/10a)
Zigui	30.83	110.97	295.50	–	47.36**	–	6.33*
Shiquan	33.05	108.27	484.90	–	22.91*	–	–
Beichuan	31.63	104.45	597.20	–	–	–	1.65*
Taibai	34.03	107.32	1543.60	–	–	–	2.13**
Mount Hua	34.48	110.08	2064.90	− 14.60**		− 11.5*	–
Diebu	34.07	103.23	2374.20	–	− 19.43*	–	–
Songpan	32.67	103.60	2881.30	7.63*	–	–	–
Hezuo	35.00	102.90	2910.00	–	–	–	1.19*

* and ** indicate that the climate trends are significant at the 0.05 and 0.01 levels, respectively, based on the Mann–Kendall test for long-term trends.

## Discussion

Our analysis confirmed that air temperature in the QDM has exhibited a trend of increase over the previous 50 years, but that the rate of temperature increase presents marked variation between different flanks and at different elevations. Overall, the rate of change of air temperature was greater on the northern flank and at higher elevations than on the southern flank and at lower elevations of the QDM in all seasons. For example, the rate of change of air temperature over the previous 50 years was as high as 0.374 and 0.456 °C/10a on the northern flank of the Qinling Mountains and at 3000–4000 m in the QDM, respectively, but as low as 0.267 and 0.303 °C/10a on the southern flank of the Qinling Mountains and at 0–1000 m in the QDM, respectively. A similar result was also identified in relation to the Qinghai–Tibet Plateau by Liu and Yao^18,19^. Additionally, research in the Tianshan Mountains, Swiss Alps, and Colorado Rockies by Xu et al.^20^, Beniston et al.^21^ and Rangwala et al.^22^, respectively, also found that the rate of climate warming in high-elevation areas has been higher than that in low-elevation areas. A possible reason is that air temperature change on northern flanks and at relatively high elevations is more sensitive to driving factors such as snow/ice coverage^22^, cloud^18,23^, water vapor^24,25^, carbon dioxide^26,27^, and soil moisture^28^.

The findings of this study highlighted the variation in air temperature increase in different seasons. In comparison with low-elevation areas, the rate of increase in air temperature was higher at high elevations in summer, autumn, and winter. Conversely, in spring, it was higher at lower elevations than at higher elevations. Dong Danhong et al.^29^ also found that the change in the rate of increase in air temperature at lower elevations was greater than that at higher elevations in spring. Possible causes of this phenomenon include human activities^30^ and solar radiation^31,32^. However, clarification of how these factors might affect the change in air temperature at different elevations in spring needs further research. The air temperature tendency on different flanks of the QDM was greater in spring and winter than in summer and autumn. The findings of this study indicated that the increase in air temperature was more pronounced in spring and winter, consistent with the results of Gao and Zhu et al. regarding air temperature change in the QDM and on the Qinghai–Tibet Plateau, respectively^8,33^. Plausible explanations for these results include the normalized difference vegetation index^34^, latent heat generated by local precipitation^35^, and air temperature inversion due to topography^36^. Anthropogenic factors such as surface albedo variation associated with land use change could explain the variation of air temperature in different seasons in mountain regions^2,37^, and the combined effects of these factors could account for the diversity of seasonal air temperature in the interior of mountain systems with complex topography.

Precipitation did not change significantly in most of the study area during 1969–2018, which is a finding consistent with the results of Wang et al. regarding the spatiotemporal changes of extreme precipitation in the North–South transition zone of China during 1960–2017^38^. However, eight weather stations did show a consistent tendency of increase in precipitation in winter. This means that the climate has become wetter in winter, which has helped alleviate winter droughts in the QDM, especially in the western QDM. Our results are in agreement with the findings of Gou Xiaoxia^30^ and Gao Xiang^8^ in relation to the northern slopes of the Tianshan Mountains and the QDM, respectively. Wetter winters might play an important role in relation to changes of vegetation, phenology^39,40^, net primary production^41^, and the normalized difference vegetation index^42^, which should be studied further in the future.

The seasonal rates of air temperature increase that differ between the various subregions and at different elevations in the QDM, will affect changes in vegetation cover and biodiversity. The rate of air temperature change was found to be greater on the northern flank than on the southern flank, and greater at higher elevations than at lower elevations. Therefore, it will result in faster increase in vegetation coverage at higher elevations on the northern flank than that at lower elevations on the southern flank, as verified by Kullman^43^ and Walther^44^. Accordingly, carbon sequestration might be greater at higher elevations than at lower elevations, which has also been verified by recent research^45^. In the future, high-elevation vegetation might migrate upward, resulting in increased species diversity.

## Conclusions

We used climate statistical analysis methods to analyze the spatiotemporal trends of air temperature and precipitation during 1969–2018 on different flanks and elevations in the QDM. The following conclusions were obtained.

1. Overall, an obvious trend of warming in different seasons has occurred over the previous 50 years in the QDM, but the rate of air temperature increase has varied markedly on different flanks and at different elevations. Precipitation has not changed significantly in most areas of the QDM during 1969 – 2018, except at eight stations that showed a significant change in precipitation to a certain degree in different seasons.
2. The seasonal rate of increase in air temperature presented notable variations on the different flanks of the QDM. Generally, the rate of air temperature change was greater on the northern flank than on the southern flank in all seasons in the QDM. Specifically, the air temperature tendency was greater in spring and winter than in summer and autumn on the different flanks in the QDM.
3. There was an increasing trend of seasonal air temperature at different elevations in different seasons in the QDM. With increasing elevation, the rate of increase in mean air temperature showed a tendency of increase in summer, autumn and winter, whereas the rate of increase in mean air temperature in spring was higher at lower elevations than at the higher elevations.

## References

[1] Zhao F, Liu JJ, Zhu WB, Zhang BP, Zhu LQ (2020). Spatial variation of altitudinal belts as dividing index between warm temperate and subtropical zones in the Qinling-Daba Mountains. J. Geogr. Sci..

[2] Zhao F, Lan XC, Li WY, Zhu WB, Li TQ (2021). Influence of land use change on the surface albedo and climate change in the Qinling-Daba mountains. Sustainability.

[3] Zhang Y (2018). Spatial variation of extreme temperature change on southern and northern slopes of Shaanxi section in Qinling Mountains during 1960–2013. Acta Geogr. Sin..

[4] Zhao F (2021). Assessing the dividing line between warm temperate and subtropical zones based on the zonality discussion on multi-dimensional response of Net Primary Productivity to climate change in the Qinling-Daba Mountains. Acta Ecol. Sin..

[5] Zhang WJ (2021). Comprehensive assessment of MODIS-derived near-surface air temperature using wide elevation-spanned measurements in China. Sci. Total Environ..

[6] Zhang B, Xu J, Wu H, Xiao F, Zhu Y (2006). Digital integration and pattern analysis of mountain altitudinal belts in China. J. Mt. Sci..

[7] Qi GZ, Bai HY, Zhao T, Meng Q, Zhang SH (2021). Sensitivity and areal differentiation of vegetation responses to hydrothermal dynamics on the northern and southern slopes of the Qinling Mountains in Shaanxi province. J. Geogr. Sci..

[8] Gao X, Bai HY, Zhang SL, He YN (2012). Climatic change tendency in Qinling Mountains from 1959 to 2009. Bull. Soil Water Conserv..

[9] Guo SZ (2020). Landscape pattern changes of woodland and grassland and its driving forces in Qinling Mountains. Acta Ecol. Sin..

[10] Li SS, Yang SN, Liu XF (2015). Spatiotemporal variability of extreme precipitation in north and south of the Qinling-Huaihe region and influencing factors during 1960–2013. Prog. Geogr.

[11] Li RK, Li YB (2021). Spatio-temporal change of soil erosion intensity on the foothills of the Daba Mountains from 1988 to 2020—A case study from the hinterland of the Three Gorges Reservoir area, China. Arab. J. Geosci..

[12] Kang MY, Zhu Y (2007). Discussion and analysis on the geo-ecological boundary in Qinling Range. Acta Ecol. Sin..

[13] Chen MR (1992). Regionalization of vertical temperature zones in QinLing Mountain range. Geogr. Res..

[14] Lei MD (1999). Vegetation of Shaanxi.

[15] Zhao F (2019). Spectra structures of altitudinal belts and their significance for determining the boundary between warm temperate and subtropical zones in the Qinling-Daba Mountains. Acta Geogr. Sin..

[16] Burn DH, Elnur MAH (2002). Detection of hydrologic trends and variability. J. Hydrol..

[17] Xu C, Li J, Zhao J, Gao S, Chen Y (2015). Climate variations in northern Xinjiang of China over the past 50 years under global warming. Quatern. Int..

[18] Liu XD, Cheng ZG, Yan LB, Yin ZY (2009). Elevation dependency of recent and future minimum surface air temperature trends in the Tibetan Plateau and its surroundings. Glob. Planet. Change.

[19] Yao YB (2016). Analysis of precipitable water vapor and surface temperature variation over Qinghai-Tibetan Plateau from 1979 to 2014. Chin. Sci. Bull..

[20] Xu M, Kang SC, Wu H, Yuan X (2018). Detection of spatio-temporal variability of air temperature and precipitation based on long-term meteorological station observations over Tianshan Mountains, Central Asia. Atmos. Res..

[21] Beniston M, Diaz H, Bradley R (1997). Climatic change at high elevation sites: An overview. Clim. Change.

[22] Rangwala I, Barsugli J, Cozzetto K, Neff J, Prairie J (2012). Mid-21st century projections in temperature extremes in the southern Colorado Rocky Mountains from regional climate models. Clim. Dyn..

[23] Beniston M, Rebetez M (1996). Regional behavior of minimum temperatures in Switzerland for the period 1979–1993. Theor. Appl. Climatol..

[24] Rangwala I, Miller JR, Xu M (2009). Warming in the Tibetan Plateau: Possible influences of the changes in surface water vapor. Geophys. Res. Lett..

[25] Rangwala I, Miller JR, Russell GL, Xu M (2010). Using a global climate model to evaluate the influences of water vapor, snow cover and atmospheric aerosol on warming in the Tibetan Plateau during the twenty-first century. Clim. Dyn..

[26] Li N (2021). Contribution of anthropogenic CO_2_ in China to global radiative forcing and its offset by the ecosystem during 2000–2015. Ann. N. Y. Acad. Sci..

[27] Cui Y, Li N, Fu Y, Chen L (2021). Carbon neutrality and mitigating contribution of terrestrial carbon sink on anthropogenic climate warming in China, the United States, Russia and Canada. J. Geogr. Sci..

[28] Rangwala I, Miller JR (2012). Climate change in mountains: A review of elevation-dependent warming and its possible causes. Clim. Change.

[29] Dong DH, Huang G (2015). Relationship between altitude and variation characteristics of the maximum, minimum temperature and diurnal temperature range in China. Chin. J. Atmos. Sci..

[30] Gou XX, Yang YH, Ye M, Zhang TG, Wang LL (2019). A study of climate change regularity of different elevations of the northern slope of Tianshan Mountain. J. Yunnan Univ. Nat. Sci. Ed..

[31] Shi S (2021). Estimation of radiative forcing and heating rate based on vertical observation of black carbon in Nanjing, China. Sci. Total Environ..

[32] Srivastava A (2020). Implications of different aerosol species to direct radiative forcing and atmospheric heating rate. Atmos. Environ..

[33] Yi Z, Fan GZ, Wei H, Wang QR (2018). Analysis of the temporal and spatial variation in land surface temperature over the Qinghai-Tibet Plateau from 1981 to 2015. J. Southwest Univ..

[34] Cui XL, Bai HY, Wang T (2013). Difference in NDVI with altitudinal gradient and temperature in Qinling area. Resour. Sci..

[35] Schneider T, O'Gorman PA, Levine XJ (2010). Water vapor and the dynamics of climate changes. Rev. Geophys..

[36] Ding RH, Xu YW, Ding X (2013). Precursor effect of terrain inversion on weather changes in Jiuhua Mountain. Meteorol. Sci. Technol..

[37] DeFries RS, Bounoua L, Collatz GJ (2002). Human modification of the landscape and surface climate in the next fifty years. Glob. Change Biol..

[38] Wang LY (2021). Spatiotemporal variations of extreme precipitation and its potential driving factors in China’s North-South Transition Zone during 1960–2017. Atmos. Res..

[39] Penuelas J (2004). Complex spatiotemporal phenological shifts as a response to rainfall changes. New Phytol..

[40] Zhang LJ, Zhu LQ, Li YH, Zhu WB, Chen YY (2022). Maxent modelling predicts a shift in suitable habitats of a subtropical evergreen tree (*Cyclobalanopsis glauca* (Thunberg) Oersted) under climate change scenarios in China. Forests.

[41] Wu G (2007). The impact of atmosphere circular system on coupling features of spring net primary productivity with precipitation in East Asia. Pak. J. Biol. Sci. PJBS.

[42] Wang J, Rich PM, Price KP (2003). Temporal responses of NDVI to precipitation and temperature in the central Great Plains, USA. Int. J. Remote Sens..

[43] Kullman L (2001). 20th century climate warming and tree-limit rise in the southern Scandes of Sweden. Ambio.

[44] Walther GR, Beissner S, Burga CA (2005). Trends in the upward shift of alpine plants. J. Veg. Sci..

[45] Zhu WB, Zhang JJ, Cui YP, Zhu LQ (2020). Ecosystem carbon storage under different scenarios of land use change in Qihe catchment, China. J. Geogr. Sci..

